# Age-dependent changes in cardiac performance, motor function, QoL, and mental status in metoprolol-treated chronic heart failure patients

**DOI:** 10.1038/s41598-018-37520-8

**Published:** 2019-01-24

**Authors:** Qiuhong Shu, Liyong Wu, Ran Zhang, Qian Zhang, Jingjing Huang, Yong Meng

**Affiliations:** Department of Cardiology, The Second Affiliated Hospital of Kunming Medical University, No. 374, Dianmian Road, Kunming, Yunnan 650101 China

## Abstract

No previous study reports the effect of age on cardiac performance, motor function and quality of life (QoL) in Chinese chronic heart failure (CHF) patients. This single-center, prospective study enrolled CHF patients with resting heart rate (RHR) > 80 bpm, who were treated with metoprolol and were followed up at 1, 3, 6, and 12 months. Changes in cardiac, motor, and QoL parameters between patients aged ≥60 years and those aged <60 years were compared at all time points. *P* < 0.05 was considered significant. A total of 154 patients were enrolled (median age: 66.39 years; 116 aged ≥60 years, 38 aged <60 years; 95% New York Heart Association class III-IV). RHR decreased significantly in both patient groups (*P* < 0.0001 for both groups). Patients aged ≥60 years had a significant improvement in both ejection fraction (EF) at 6 and 12 months and in cardiac index (CI) at 3, 6, and 12 months. However, no major difference was observed in motor function in both groups. Significantly higher SF-8 scores showed greater improvement in QoL in the <60 age group at 12 months (*P* = 0.0008). Metoprolol demonstrated improvement in cardiac performance, motor function, QoL, and anxiety with increase in depression and burnout in both genders; however, the findings were independent of age.

## Introduction

Chronic heart failure (CHF) is a complex physiological syndrome caused from structural or functional alterations to the myocardium^1^. The prognosis of CHF is closely associated with age >40 years, comorbid conditions, and oxygen consumption^2,3^. Further, CHF significantly impairs the patients’ ability to perform daily activities^4,5^ along with causing multiple neurological disorders including stroke and cognitive impairment^6^. Approximately, 4.2 million people are currently affected by heart failure (HF) in China, which may be attributed to rapid change in lifestyle and industrialization^7^. These numbers are expected to rise further due to increasing proportion of aging population and other risk factors^8^. Despite advancements, the prognosis of HF patients have been reportedly poor^9^ and requires additional palliative care along with symptomatic control among the elderly patients with CHF^10^.

β-Adrenoceptors are essential regulators of cardiovascular homeostasis. Types of β-adrenoceptors, namely, β-1 and β-2, are predominantly present in the heart and vascular smooth muscles, respectively. The inhibitors of β-adrenoceptors, that is β-blockers, possess vasodilating effects, which are mediated by α-1 adrenoceptor blockade, β-2 adrenoceptor agonism, or nitric oxide synthesis. Also, β-blockers are competitive inhibitors of β-receptors and tend to inhibit the effects of catecholamine in CHF^11,12^. Thus, β-blockers are one of the mainstays of treatment for CHF because of their ability to reverse the neurohumoral effects of the sympathetic nervous system^13^, with ensuring symptomatic benefits by lowering heart rate (HR) and contractility, consequently lowering the mortality of CHF^2,14^. The same has been demonstrated in terms of improving survival^15–17^ and quality of life (QoL) of CHF patients, indicating their importance in CHF management^18–21^. On the contrary, administration of β -blockers increases the risk of depression^22^, a key factor for deteriorating QoL and increased mortality risk among CHF patients^23^.

According to the MERIT-HF^15,24^ and RESOLVD studies^24^, metoprolol succinate, a cardioselective β-blocker, is one of the β-blockers recommended for preventing the incidence of death in HF patients^2^. Additionally, CHF patients reported improvement in QoL in terms of physical activity, social functions and life satisfaction compared with standard therapy^21^ and placebo^25^. Previously, only one study has reported the overall effect of metoprolol on cardiac performance, motor function, and QoL in Chinese patients^26^ with no separate evaluation in elderly patients. Thus, indicating the paucity of data on age-dependent cardiac, motor, and QoL outcomes of metoprolol. The present study will add insight about the risk-to-benefit ratio of metoprolol treatment in aged population. Therefore, this study was performed to compare the effect of metoprolol treatment in elderly (aged ≥60 years) and non-elderly Chinese CHF patients (aged <60 years) in terms of change in cardiac function, motor function, QoL, and mental status.

## Results

### Baseline characteristics

**Figure 1:**
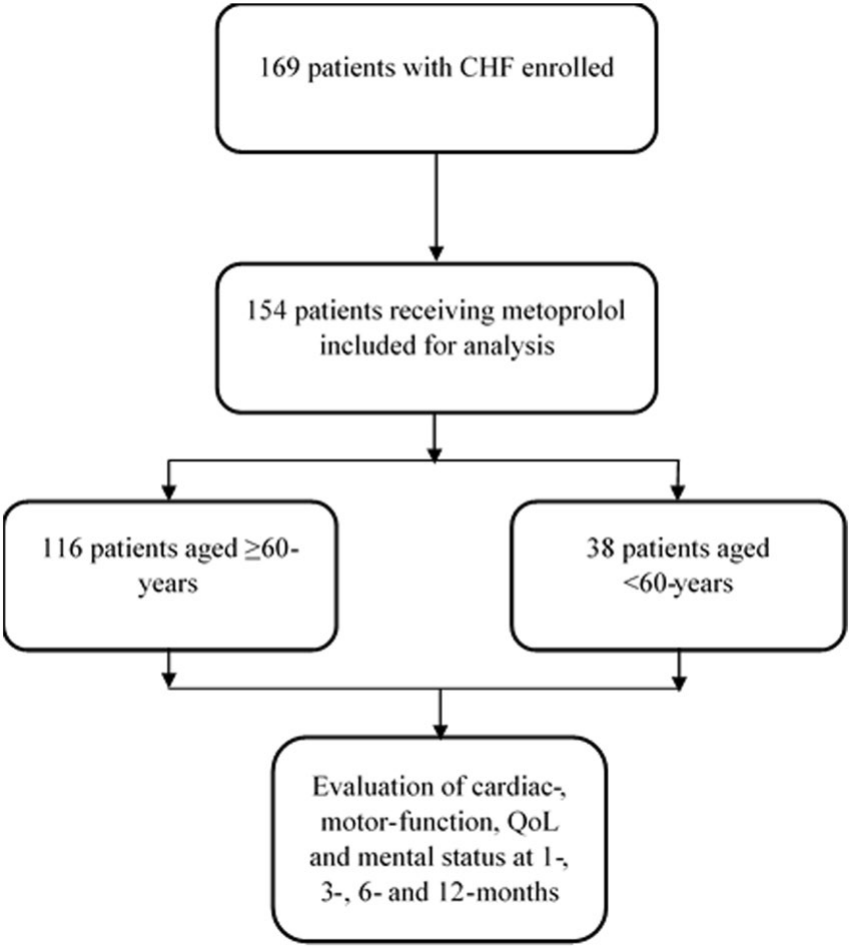
Study flow.

A total of 154 patients (median age 66.39 years; body mass index 23.85 ± 3.62 kg/m^2^; 65.5% male patients) were analyzed of the 169 patients included. Of the 154 patients, 116 were elderly (aged ≥60 years) and the remaining 38 patients were non-elderly (aged <60 years) (Fig. 1). At baseline, family history of cardiac disease and hypertension were reported in 35.0% and 74.6% patients, respectively. Approximately, 95% of patients were categorized as New York Heart Association (NYHA) class III-IV. Other important comorbidities and disease history are presented in Table 1. At baseline, patients aged ≥60 years had significantly higher HR (87.03 ± 4.53 vs. 81.31 ± 6.75, respectively, *P* < 0.0001) and systolic blood pressure (SBP) (132.81 ± 6.55 vs. 124.74 ± 14.75 respectively, *P* = 0.0014) compared to patients aged <60 years.

**Table 1:** Patient baseline characteristics.

Characteristic	N (%)
Number of patients	154 (100)
≥60 years	116 (75.34)
<60 years	38 (24.66)
Age (years), median	66.39
Gender	
Male	101 (65.58)
Female	53 (34.41)
BMI (kg/m^2^), mean (SD)	23.85 (3.62)
Glomerular filtration rate (mL/min/1.73 m^2^), mean (SD)	73.9 (26.8)
Disease history/Comorbidity	
Hypertension	115 (74.67)
Type 2 diabetes	101 (65.58)
Coronary artery disease	99 (64.28)
Stroke	137 (88.96)
Family history of cardiac disease	54 (35.06)
Smoker	111 (72.07)
Alcohol consumption	86 (55.84)
Myocardial infarction	59 (38.31)
NYHA class III-IV	145 (94.15)
Drug usage history	
ACEIs/ARBs	150 (97.40)
Diuretic	145 (94.15)
Digoxin	114 (74.02)
Antithrombotic drugs	146 (94.80)

### Reduction in SBP and HR

During the 1-year period, patients with higher SBP and HR were treated with an average metoprolol dose of 99.75 mg. Overall resting heart rate (RHR) as measured by resting electrocardiogram decreased significantly from a baseline value of 82.72 ± 6.73 to 64.86 ± 3.21 bpm at 12 months (*P* < 0.0001). Similarly, there was a significant drop in SBP from a baseline value of 126.73 ± 13.64 to 123.35 ± 12.31 (*P* = 0.0230) at the 1^st^ month follow-up, followed by an increase (124.70 ± 9.67) at the 3^rd^ month follow-up and 125.11 ± 6.67 at the 6^th^ month follow-up. At the 12^th^ month follow-up, SBP levels reached a stable level of 123.37 ± 6.88 mmHg (*P* = 0.0067), which was comparable with the levels observed at the 1^st^ month following metoprolol treatment.

Decrease in HR from baseline to 12 months was significant in patients aged <60 years (87.03 ± 4.53 vs. 65.58 ± 3.08, *P* < 0.0001) and ≥60 years (81.31 ± 6.75 vs. 64.29 ± 6.75, *P* < 0.0001); however, SBP levels decreased significantly only in patients aged <60 years (*P* < 0.0001). Although at 12 months, there was greater decrease in HR and SBP in patients aged < 60 years, this group had significantly higher HR (65.58 ± 3.08 vs. 64.29 ± 6.75, *P* = 0.0319) and similar SBP (124.74 ± 3.22 vs. 122.93 ± 6.62, *P* = 0.1071) compared with patients aged ≥60 years.

### Improvement in cardiac performance, motor function, and QoL: a comparison

Among the cardiac function parameters, EF decreased non-significantly from baseline to 1 and 3 months in patients aged <60 years (37.08% ± 6.18% to 34.50% ± 6.44%; *P* = 0.0791 and 36.47% ± 5.57%; *P* = 0.655, respectively). Decrease in EF from baseline to 1 and 3 months was significant in patients aged ≥60 years (37.79% ± 5.89% to 35.25% ± 6.08%; *P* = 0.014 and 35.46% ± 4.92%; *P* < 0.0001, respectively). However, treatment with metoprolol significantly improved EF at 6 and 12 months in both the age groups (*P* < 0.0001 for change, Table 2). For cardiac index (CI), a non-significant decrease was observed from baseline to 1 month in patients aged <60 years, whereas the decrease was significant in patients aged ≥60 years (1.79 ± 0.21 vs. 1.71 ± 0.27, *P* = 0.015). A similar trend of overall significant increase in CI was reported from baseline to 3, 6, and 12 months in patients aged ≥60 years (1.79 ± 0.21 vs. 2.24 ± 0.19, 2.61 ± 0.18 and 2.78 ± 0.25, *P* < 0.0001 for all comparisons). CI was similar for both the groups at all follow-up durations, showing similar improvement in cardiac function in elderly and patients aged <60 years after metoprolol treatment (Table 2).

**Table 2:** Comparison of cardiac function, motor function, and QoL.

Time	Cardiac Function					
	EF (%)			CI (L/min^*^m^2^)		
	≥60	<60	*P* Value	≥60	<60	*P* Value
Baseline	37.79 ± 5.89	37.08 ± 6.18	0.5190	1.79 ± 0.21	1.76 ± 0.23	0.4566
1 month	35.25 ± 6.08^#^	34.50 ± 6.44	0.5164	1.71 ± 0.27^##^	1.77 ± 0.24	0.2242
3 months	35.46 ± 4.92^#^	36.47 ± 5.57	0.2897	2.24 ± 0.19	2.30 ± 0.18	0.2558
6 months	47.95 ± 4.35^*^	47.55 ± 4.95^*^	0.4000	2.61 ± 0.18^*^	2.61 ± 0.18	1.000
12 months	50.4 ± 4.35^*^	49.65 ± 4.25^*^	0.3362	2.78 ± 0.25^*^	2.72 ± 0.23^*^	0.1926
	**6MWT (m)**			**VSAQ (score)**		
Baseline	369.37 ± 33.84	367.10 ± 34.69	0.7168	6.49 ± 1.04	6.64 ± 1.18	0.4568
1 month	344.51 ± 32.23^##^	331.73 ± 32.08^##^	**0.0353**	4.96 ± 0.88^##^	4.79 ± 0.89^##^	0.3043
3 months	352.45 ± 34.03^#^	345.55 ± 29.911^##^	0.2661	5.44 ± 0.98^##^	5.79 ± 1.14^##^	0.0687
6 months	399.41 ± 21.64^*^	395.73 ± 21.43^*^	0.3632	7.87 ± 1.04^*^	7.80 ± 1.07^*^	0.7212
12 months	416.32 ± 21.35^*^	416.81 ± 19.26^*^	0.9002	8.38 ± 0.93^*^	8.21 ± 1.04^*^	0.3439
	**SF-8**			**MLHFQ**		
Baseline	43.96 ± 2.75	44.18 ± 2.60	0.6652	74.20 ± 3.37	74.02 ± 4.00	0.7856
1 month	39.37 ± 1.66^##^	39.42 ± 1.64^##^	0.8718	88.56 ± 4.42^##^	89.52 ± 4.14^##^	0.2399
3 months	42.36 ± 2.57^##^	42.37 ± 3.11^##^	0.9843	86.37 ± 5.09^##^	88.55 ± 4.64^##^	**0.0206**
6 months	48.81 ± 1.22^*^	49.05 ± 1.18^*^	0.2904	64.28 ± 3.53^*^	65.18 ± 4.71^*^	0.2131
12 months	51.83 ± 2.26^*^	52.89 ± 1.67^*^	**0.0086**	53.90 ± 8.42^*^	53.40 ± 7.20^*^	0.7429

^*^P < 0.0001 significant increase compared with baseline.

^#^*P* < 0.01, ^##^*P* < 0.0001 significant decrease from baseline.

Motor function evaluation with 6-minute walk test (6MWT) showed that patients in the ≥60- and <60-year age groups walked similar distances at all the follow-up points. At the 1-month follow-up, patients aged ≥60 years had significantly higher walking distance on the 6MWT compared with patients aged <60 years (344.51 ± 32.23 vs. 331.73 ± 32.08, *P* < 0.0353). However, patients aged <60 years had non-significantly higher motor function compared with patients aged <60 years at 12 months (416.81 ± 19.26 vs. 416.32 ± 21.35, *P* = 0.9002, Table 2). Within the ≥60-year age group, a trend of initial decrease in motor function from baseline to up to 3 months was observed; however, the motor function on 6MWT significantly increased at 6 and 12 months compared with baseline (*P* < 0.0001 for both comparisons, Table 2). Veterans Specific Activity Questionnaire (VSAQ) scores were similar in both the groups at all the time points, indicating no significant differences in motor function. Similar to 6MWT, a decreasing trend was seen in VSAQ scores from baseline to up to 3 months followed by a significant improvement (Table 2).

A comparison of QoL between the two groups via short form-8 questionnaire (SF-8) scales showed no significant difference in scores until 6 months; however, at the 12-month follow-up, patients aged <60 years had significantly higher scores than those aged ≥60 years, indicating a greater improvement in QoL (52.89 ± 1.67 vs. 51.83 ± 2.26, *P* = 0.0086). In both the age groups, the SF-8 scores decreased initially until 3 months *(P* < 0.0001 for both), followed by a significant improvement at 6 and 12 months compared with baseline (*P* < 0.0001 for both, Table 2). Compared to baseline, a significant improvement in the Minnesota Living with Heart Failure questionnaire (MLHFQ) scores at 6 and 12 months was seen (*P* < 0.0001), which was preceded by worsening of QoL at 1 and 3 months. At 3 months, patients aged ≥60 years had significantly higher MLHFQ scores than those aged <60 years (86.37 ± 5.09 vs. 88.55 ± 4.64, *P* = 0.0206), with similar scores observed at all other time points (Table 2).

### Change in mental status

**Figure 2:**
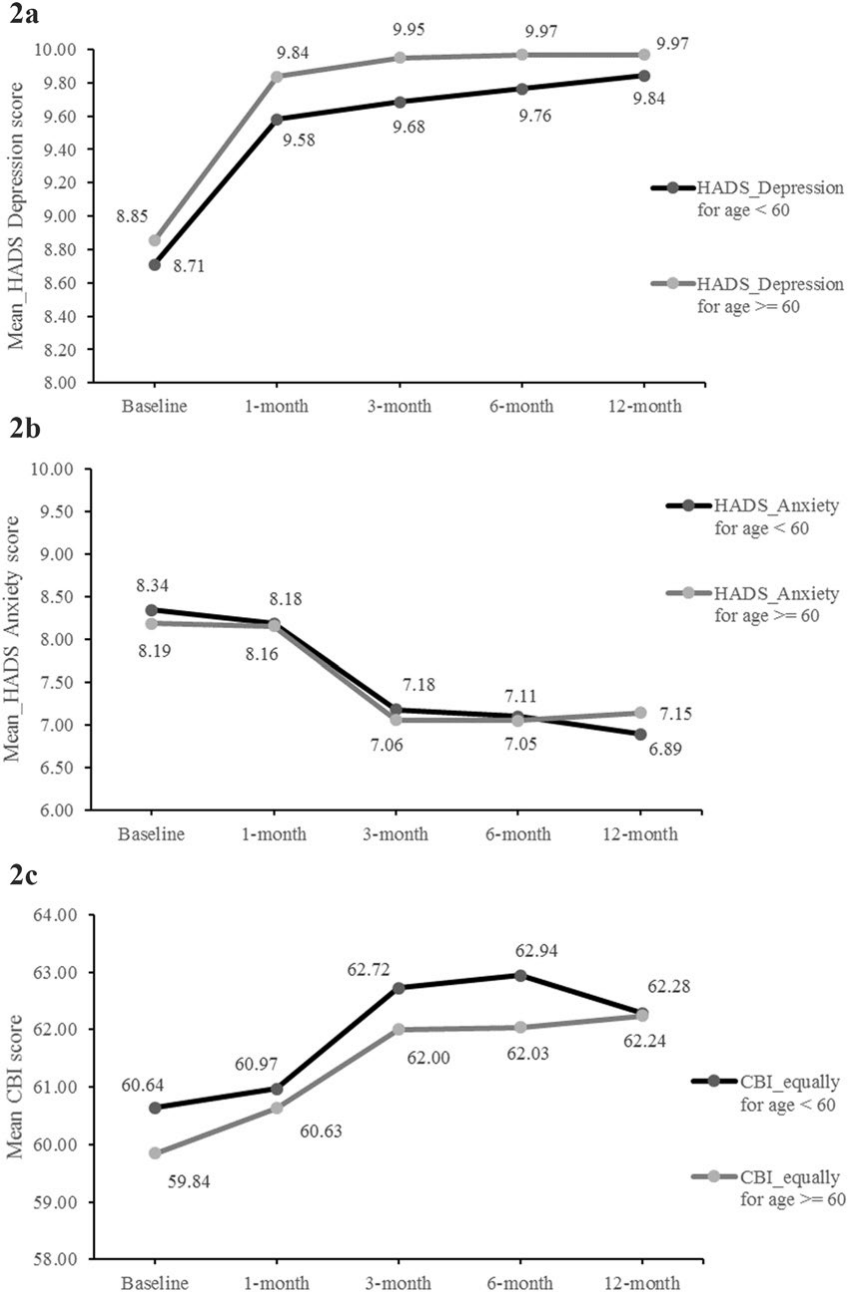
(**a**) Change in HADS depression and anxiety – Depression (**b**) Change in HADS depression and anxiety – Anxiety (**c**) Change in combined CBI scores – Burnout.

Among the CHF patients aged ≥60 years, the Hospital Anxiety and Depression Scale (HADS) depression score was higher throughout the study duration compared with patients aged <60 years, with no significant difference in the depressive symptoms reported at any of the follow-up visits (Fig. 2a). However, the depression score worsened significantly from baseline to 1 (*P* = 0.0092), 3 (*P* = 0.0039), 6 (*P* = 0.0029), and 12 months (*P* = 0.0023) in patients aged ≥60 years. Although the depression scores worsened in patients aged <60 years from baseline to 1 (*P* = 0.1530), 3 (*P* = 0.1152), 6 (*P* = 0.0832), and 12 months (*P* = 0.0612), the change was not significant. Figure 2b presents the HADS anxiety scores, which were consistently lower in the older patients till 6 months compared with patients aged <60 years, with higher scores observed at 12 months. In the patients aged ≥60 years, HADS anxiety scores were consistent till 1 month (*P* = 0.9125) but decreased significantly at 3, 6, and 12 months from baseline (*P* < 0.0001 for all comparisons). The decrease in HADS anxiety scores in patients aged <60 years followed a similar trend, with non-significant decrease till 1 month from baseline (*P* = 0.7372) followed by significant decrease at 3 (*P* = 0.0039), 6 (*P* = 0.0019), and 12 months (*P* = 0.0002).

Compared with patients aged <60 years, mean Copenhagen Burnout Inventory (CBI) scores in the patients aged ≥60 years was consistently and non-significantly lower at baseline to 12 months (*P* = 0.3120, *P* = 0.8046, *P* = 0.6684, *P* = 0.5897, and *P* = 0.9812 at baseline, 1, 3, 6, and 12-months, respectively) (Fig. 2c). Within the patient group aged ≥60 years, overall CBI scores worsened significantly at 3 (*P* = 0.0127), 6 (*P* = 0.0166), and 12 months (*P* = 0.0062) from baseline. On the other hand, the CBI scores increased in the patients aged <60 years; however, the increase was non-significant.

Age at baseline was not associated with greater risk of depression, i.e. patients aged ≥60 years had similar odds of experiencing depression compared with patients aged <60 years (odds ratio [OR] 1.03, 95% confidence interval (CI) 0.4960–2.1526, *P* = 0.93). However, male patients had significantly greater odds of depression compared with female patients (OR 2.0621, 95% CI 1.0494–4.0519, *P* = 0.0357). In the subgroup analysis, except significant greater CI in the HADS score ≥10 group versus the HADS score ≤11 group (*P* = 0.0421), no significant differences were observed in mean EF, CI, 6MWT, and VSAQ (Supplementary Table 1). However, a significant change in the variables within the group was seen at different time points.

## Discussion

We explored the impact of metoprolol treatment on cardiac, motor, and QoL outcomes in CHF patients and reported improvement in all the aspects^26^. Since β-blockers are known to worsen psychological conditions, physicians must rightly choose their patients to treat CHF without deteriorating their QoL and mental status. To the best of our knowledge, this is the first study to compare the cardiac, motor, QoL, and mental/neurological outcomes in patients with CHF. An expected and significant reduction in HR was observed in both the groups from baseline to 12 months, due to the cardioselective action of β1-blockers^27^. In our study, patients aged ≥60 years did not achieve significant SBP reduction, which was in consistence with previous study by Holfler and Morgenstern, where the magnitude of blood pressure (BP) reduction was similar in all the groups without any age-dependent changes^28^. In our study, SBP was observed to be decreased significantly at the 1-month follow-up. However, very minor but consistent increase in SBP was observed till the 12-month follow-up. This is contrary to the previous observations in which no significant change in SBP was observed at 1 month^29^ and comparable decrease in SBP was observed after 3 months^30^. A possible reason for this slight increase in SBP in our study could be explained by correlating aging with HF. An increase in the levels of circulating catecholamines has been observed both in CHF and aged individuals along with desensitization of the β-adrenoceptors due to decrease in their number^31,32^. Additionally, a disturbed β-adrenoceptor-mediated vasodilation has also been associated with aging due to hyper-expression and increased activity of G protein coupled receptor kinase (GRK2) in the vascular system with advancing age^33^.

With respect to cardiac performance, a biphasic response was observed wherein initial decrease till 1 month was followed by a significant improvement in EF and cardiac index until 12 months in both the age groups and no significant difference was seen when compared between the groups. This confirmed the non–age-dependent action of the β1-blocker, metoprolol on both EF and CI^28^. Further evidence suggests the benefits of β-blocker use in CHF patients with reduced Left ventricular ejection fraction (LVEF)^34^ and preserved LVEF^35^. Similar to our findings, a study by Neto *et al*. reported improved EF, lowered LVEF, and cardiac frequency in patients with NYHA II-IV grade CHF^36^. β1-blockers are also known to demonstrate improvement in cardiac index by 0.3 to 0.8 L/min*m^2^ due to multiple mechanisms^37^. In our study, the magnitude of cardiac index change was 0.99 L/min*m^2^ in patients aged ≥60 years and 0.96 L/min*m^2^ in patients aged <60 years, showing overall a similar effect in both age groups and concordance with previous findings.

The trend of motor improvement was accompanied with a decreasing pattern in motor function in both age groups from baseline to 1 month, followed by an improvement till the end of follow-up. The decrease in 6MWT distance and VSAQ scores in this study may be correlated with decreased cardiac performance at 1 month as patients with CHF usually report myopathy of both cardiac and skeletal muscles^38^. This further validates the finding that cardiovascular diseases are associated with deteriorating motor functions^39,40^. 6MWT is a reliable measure for motor function in multiple studies which have shown a significant improvement in CHF patients^41,42^. Similar to previous findings, in the current study, 6MWT predicted a significant improvement in motor function. However, the improvement was in both groups without any significant difference among the patients of different ages. Similarly, VSAQ has been validated in Chinese patients with HF^43^ and has further shown to be associated with poor QoL on MLHFQ scores^44^. QoL in patients with CHF is significantly low due to the effect of HF on the motor functions, constant requirement for healthcare support, which is a burden for patients with lower socioeconomic status^45^. β-blockers, including metoprolol, however have reported a significant improvement in the QoL in CHF patients using SF-36, MLHFQ, and Quality of Life in chronic Heart Failure Questionnaire (QLQ-CHF)^21,25^. However, a recent randomized trial by Mittal *et al*. showed that metoprolol did not significantly improve the QoL in CHF patients using the SF-36 questionnaire^46^. In another study by Waagstein *et al*., metoprolol treatment significantly improved HR, cardiac function, motor function, and QoL at 12 months^47^. In our study, the trend of QoL in CHF patients after metoprolol treatment was similar to the cardiac function and motor function trend, showing an initial decline in QoL till 1 month with a subsequent improvement till 12 months. Similar trend of change in cardiac, motor function, and QoL indicated a relation between them.

Although CHF patients are susceptible for developing depression and anxiety due to neurohormonal dysregulation^48^, these symptoms are mostly underestimated leading to progression of comorbidities^49^, thereby increasing the risk of mortality^50,51^. There is mounting evidence supporting the fact that depression and anxiety are highly prevalent in elderly patients impacting their QoL^52,53^. From our previous experience, worsening of in patients with CHF, which was non-related to metoprolol, induced HR reduction but possibly due to administration of other CV drugs or due to involvement of metabolizing enzymes. Further the anxiolytic effect of metoprolol was observed in CHF patients via RHR reduction^54^. In the present study, patients aged ≥60 years reported consistent and non-significant higher HADS_depression score after metoprolol treatment. This corroborates the findings that metoprolol and other β-blockers are associated with depression^22,55^ and the dosage and duration of treatment should be carefully monitored in all patients irrespective of their age. Owing to its direct effects on the central β-receptors^56^, treatment with metoprolol effectively controlled anxiety in both the groups resulting in similar HADS_anxiety scores throughout the study. Evaluation of burnout in CHF patients aged ≥60 years showed significant worsening from baseline to end of follow-up with no significant difference seen compared with the <60 year age group. Since older patients had lower burnout throughout the study and no previous findings have elucidated the difference in patients of different age groups, further studies are warranted to explore the burnout using CBI scores.

The study has some major strengths, which include (i) first study to determine the age-dependent changes in CHF patients and (ii) the study included a comprehensive evaluation of cardiac, functional, motor, and mental effects in the patients after metoprolol administration. In addition, our study also had a few limitations due to which the results must be carefully interpreted: (i) the study had only the treatment group and no placebo or active control (β-blocker effective in CHF: carvedilol or bisoprolol) group was included, presence of which could have projected the efficacy of metoprolol in a more effective manner and (ii) the subjective questionnaires could have induced a factor of bias to the findings (however, this was addressed to an extent by the provision of assistance by healthcare practitioners (HCPs) for responding to the questions); (iii) since depression and burnout in HADS_depression and CBI scores worsened, a longer follow-up could have helped in determining the extent of change depending on the duration of therapy; (iv) known history (duration, severity and any other treatments) would also yield more reliable results; and (v) the patients in the <60- and ≥60-year age groups were distributed in 1:3 proportion. Therefore, due to a small sample size of patients aged <60 years (n = 38), the conclusions drawn should be carefully extrapolated as the study might be inadequately powered. Larger randomized studies shall be performed to confirm the findings of this study.

## Materials and Methods

### Study design and patient population

Complete study design and criteria for including participants have been described elsewhere^26^. Briefly, this prospective study enrolled CHF patients (HR > 80 bpm) with or without neuropsychiatric disorders treated at The Second Affiliated Hospital of Kunming Medical University between February 2013 and April 2016. Patients were excluded if they had (i) HR < 60 bpm; (ii) SBP < 90 mmHg; (iii) history of metoprolol use in the last 3 months; (iv) <6 months of expected survival; (v) pacemaker implanted; (vi) contraindication to β-blockers; (vii) been administered class I or class III anti-arrhythmic drugs; (viii) recent heart attack; and (ix) undergone coronary bypass surgery. The study complied with Good Clinical Practices, declaration of Helsinki and its subsequent revisions. All the patients were included in the study only after obtaining a signed informed consent. All the experimental protocols were approved by The Second Affiliated Hospital of Kunming Medical University licensing committee and the study was conducted in accordance with relevant guidelines and regulations.

### Treatment plan

Data collection was performed for patients with history of initial metoprolol dosing of 23.75 or 47.5 mg (Betaloc^®^ ZOK, AstraZeneca, Sweden). The dose was subsequently increased by 23.75 mg/week until target HR of 60–70 bpm was achieved in few patients during follow-up.

### Study outcomes and measurements

The study outcome was to compare cardiac function, motor function, QoL, and mental status at 1, 3, 6, and 12-months from baseline in elderly patients aged ≥60 years versus patients aged <60 years. A trend of change in all the outcomes was also evaluated within the groups.

The measurement scales and method for assessment of SBP, RHR, cardiac function (EF and CI), motor function (6MWT and VSAQ), and QoL (SF-8 and MLHFQ) are described elsewhere^26^. Mental assessment was performed using the HADS and CBI scale. HADS is a self-administered questionnaire, with seven questions each for depression and anxiety. Patients with HADS score 8–10 and 11–21 were considered borderline normal and abnormal, respectively^57^. The CBI questionnaire measures personal, work-, and client-related burnout by patients’ perspective^58^ Patients.

who experienced difficulty in completing the HADS and CBI questionnaires were assisted by the HCPs. A subgroup analysis was performed to compare the EF, CI, 6MWT, and VSAQ between patients with HADS_depression score ≥11 versus HADS_depression score ≤10 at baseline. The subgroup analysis was performed irrespective of the age of the patients.

### Statistical analysis

Descriptive statistics was used to present the baseline characteristics as mean ± standard deviation (SD), median (range), numbers, and percentages. Values for EF, CI, 6MWT, VSAQ, SF-8, MLHFQ, HADS, and CBI in both age groups and genders were presented as mean ± SD. Student’s t-test was used to compare the mean values for all the parameters between the two groups. A *P* value of <0.05 was considered statistically significant.

## Conclusion

Treatment with metoprolol demonstrated an effective improvement in cardiac performance, motor function, QoL, and anxiety in patients from both the groups, whereas an increase in the depression and burnout was noted. Despite the changes within the groups, the patients belonging to both ≥60-year and <60-year age groups had similar changes after treatment, indicating the consistent efficacy of metoprolol for CHF patients irrespective of the age.

## Supplementary information

Related Manuscript File
