# Supplementary material for: Age-dependent changes in cardiac performance, motor function, QoL, and mental status in metoprolol-treated chronic heart failure patients
Source: Sci Rep. 2019 Jan 24;9:453. doi: 10.1038/s41598-018-37520-8 (PMC6345765; doi:10.1038/s41598-018-37520-8)
Supplement: Supplementary file 1 — Related Manuscript File [file 41598_2018_37520_MOESM1_ESM.docx]

**Age-dependent changes in cardiac performance, motor function, QoL, and mental status in metoprolol-treated chronic heart failure patients**

Qiuhong Shu^1^, Liyong Wu^1^, Ran Zhang^1^, Qian Zhang^1^, Jingjing Huang^1^, Yong Meng^1*^

**^1^**Department of Cardiology, The Second Affiliated Hospital of Kunming Medical University, No. 374, Dianmian Road, Kunming, Yunnan, China, 650101.

**Supplementary Tables**

**Supplementary Table 1. Comparison of cardiac and motor indicators in patients with HADS_depression scores ≥11 versus ≤10**

| **Time** | **Cardiac Function** | | | | | |
| --- | --- | --- | --- | --- | --- | --- |
|  | **EF (%)** | | | **CI (L/min*m^2^)** | | |
|  | **≥11*** | **≤10*** | ***P* Value** | **≥11*** | **≤10*** | ***P* Value** |
| Baseline | 37.85 ± 5.86 | 37.41 ± 5.59 | 0.7288 | 1.77 ± 0.20 | 1.78 ± 0.20 | 0.8215 |
| 1 month | 34.39 ± 5.86^$$^ | 35.29 ± 6.11 | 0.4980 | 1.64 ± 0.30^$$^ | 1.76 ± 0.22 | **0.0421** |
| 3 months | 35.80 ± 5.28 | 34.59 ± 4.46 | 0.2656 | 2.29 ± 0.22^$^ | 2.23 ± 0.19^$^ | 0.1901 |
| 6 months | 47.61 ± 4.99^#^ | 48.44 ± 4.28^#^ | 0.4000 | 2.63± 0.17^#^ | 2.61 ± 0.19^#^ | 0.6168 |
| 12 months | 47.22 ± 3.82^##^ | 48.83 ± 4.28^##^ | 0.0761 | 2.65 ± 0.26^##^ | 2.64 ± 0.26^##^ | 0.8622 |
|  | **Motor Function** | | | | | |
|  | **6MWT (m)** | | | **VSAQ (score)** | | |
| Baseline | 366.66 ± 35.43 | 368.44 ± 33.82 | 0.8166 | 6.23 ± 1.00 | 6.68 ± 1.09 | 0.6698 |
| 1 month | 340.12 ± 33.00^$$^ | 342.49 ± 30.22^$$^ | 0.7354 | 4.82 ± 0.81^$$^ | 4.94 ± 0.90^$$^ | 0.5275 |
| 3 months | 351.07 ± 36.13^∆^ | 346.80 ± 33.82^∆^ | 0.5822 | 5.54 ± 0.98^∆^ | 5.52 ± 0.99^∆^ | 0.9270 |
| 6 months | 395.32 ± 21.92^#^ | 399.46 ± 21.06^##^ | 0.3858 | 7.80 ± 1.06^#^ | 7.96 ± 1.03^#^ | 0.4902 |
| 12 months | 405.27 ± 17.93^##^ | 407.07 ± 20.09^##^ | 0.6698 | 7.90 ± 0.80^##^ | 7.98 ±0.99^##^ | 0.6678 |
|  |  |  |  |  |  |  |

** 41 patients were included in both groups.*

*^$$^ Significant decrease in value from baseline to 1 month within same group.*

*^$^ Significant improvement from baseline to 3 months within same group.*

*^#^ Significant improvement from baseline to 6 months within same group.*

*^##^ Significant improvement from baseline to 12 months within same group.*

*∆ Significant decrease from baseline to 3 months within same group.*
